# Author Correction: Vps3 and Vps8 control integrin trafficking from early to recycling endosomes and regulate integrin-dependent functions

**DOI:** 10.1038/s41467-021-25201-6

**Published:** 2021-09-29

**Authors:** Caspar T. H. Jonker, Romain Galmes, Tineke Veenendaal, Corlinda ten Brink, Reini E. N. van der Welle, Nalan Liv, Johan de Rooij, Andrew A. Peden, Peter van der Sluijs, Coert Margadant, Judith Klumperman

**Affiliations:** 1 Section Cell Biology, Center for Molecular Medicine, University Medical Center Utrecht, Utrecht University, Heidelberglaan 100, 3584 CX Utrecht, The Netherlands; 2 Section Molecular Cancer Research, Center for Molecular Medicine, University Medical Center Utrecht, Utrecht Universty, Heidelberglaan 100, 3584 CX Utrecht, The Netherlands; 3 Department of Biomedical Science, The University of Sheffield, Sheffield, S10 2TN UK; 4 Department of Molecular Cell Biology, Sanquin Research, Plesmanlaan 125, 1066 CX Amsterdam, The Netherlands; 5 Present Address: Department of Ophthalmology, Weill Cornell Medicine, 1300 York Ave, New York, NY 10065 USA; 6 Present Address: UCL Cancer Institute, University College London, 72 Huntley Street, London, WC1E 6DD UK; 7 Present Address: Cellular Protein Chemistry, Bijvoet Center for Biomolecular Research, Utrecht University, 3584 CH Utrecht, The Netherlands

**Keywords:** Integrins, Transport carrier, Endosomes

Correction to: *Nature Communications* 10.1038/s41467-018-03226-8, published online 23 February 2018.

The original version of this Article contained an error in the spelling of the authors Caspar T.H. Jonker, Romain Galmes, Tineke Veenendaal, Corlinda ten Brink, Reini E.N. van der Welle, Nalan Liv, Johan de Rooij, Andrew A. Peden, Peter van der Sluijs, Coert Margadant and Judith Klumperman, which were incorrectly given as C.T.H. Jonker, R. Galmes, T. Veenendaal, C. ten Brink, R.E.N. van der Welle, N. Liv, J. de Rooij, A.A. Peden, P. van der Sluijs, C. Margadant and J. Klumperman. This has now been corrected in both the PDF and HTML versions of the Article.

