# Supplementary material for: Vps3 and Vps8 control integrin trafficking from early to recycling endosomes and regulate integrin-dependent functions
Source: Nat Commun. 2018 Feb 23;9:792. doi: 10.1038/s41467-018-03226-8 (PMC5824891; doi:10.1038/s41467-018-03226-8)
Supplement: Supplementary file 1 — Supplementary Information [file 41467_2018_3226_MOESM1_ESM.pdf]

Vps3 and Vps8 control integrin trafficking from early to recycling endosomes and regulate integrin-dependent functions

Jonker CTH *et al.*

Supplementary information

Supplementary Figure 1

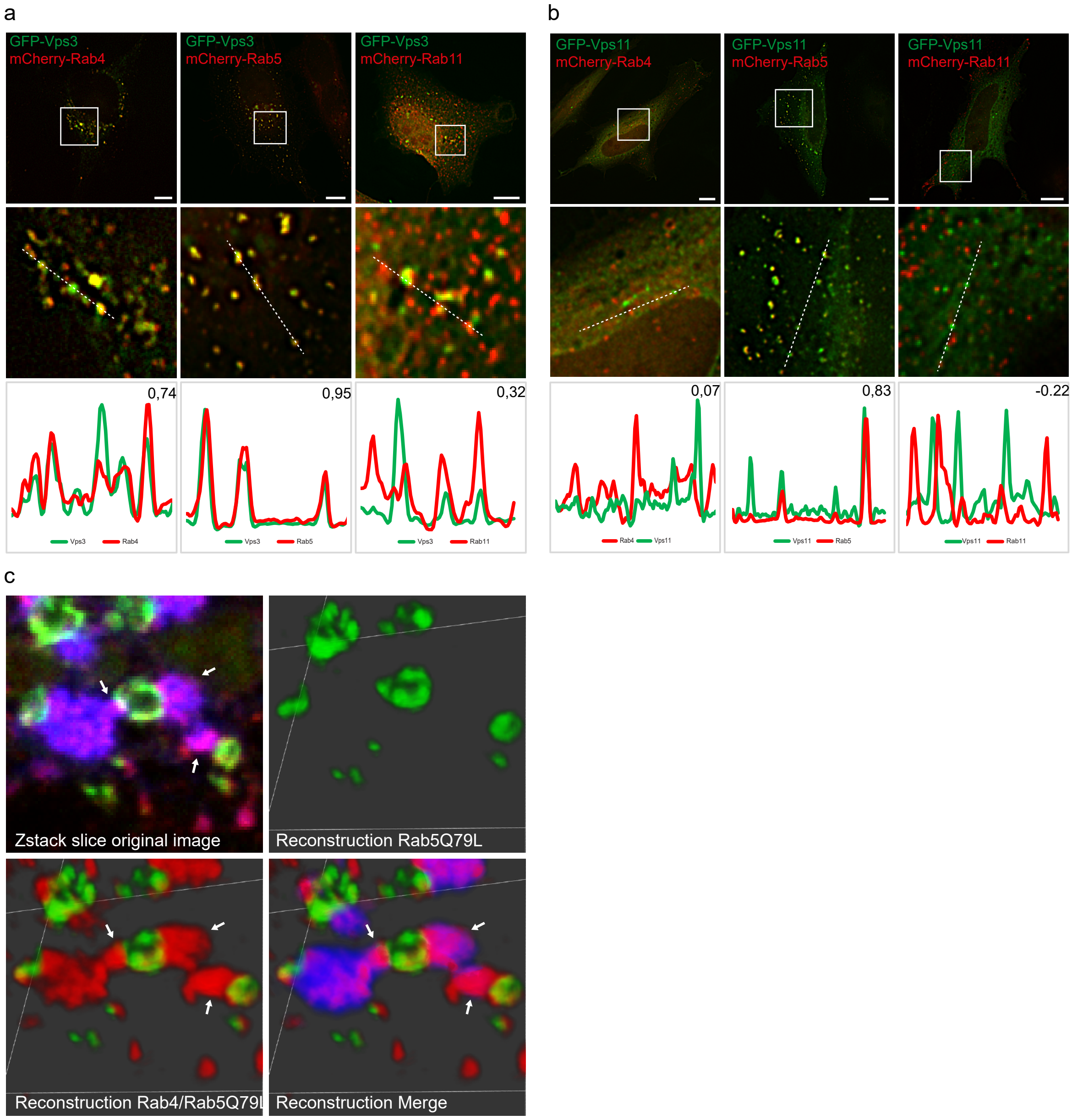

Supplementary Figure 1. (a) HeLa cells expressing GFP-Vps3 together with mCherry-Rab4, mCherry-Rab5, or mCherry-Rab11. GFP-Vps3 co-localizes strongly with Rab4 and Rab5 and mildly with Rab11. (b) HeLa cells expressing GFP-Vps11 together with mCherry-Rab4, mCherry-Rab5 or mCherry-Rab11. GFP-Vps11 co-localizes strongly with Rab5 but not with Rab4 or Rab11. (c) IF of HeLa cells expressing Rab5Q79L-GFP, Rab4-FLAG and Vps8-V5. Vps8 localizes to Rab4 positive patches on Rab5Q79L positive enlarged endosomes. Three-dimensional reconstruction of the Z-stack shows that Rab4 and Vps8 overlap and are connected to the Rab5Q79L positive endosome (arrows). Bar, 10µm.

Supplementary Figure 2

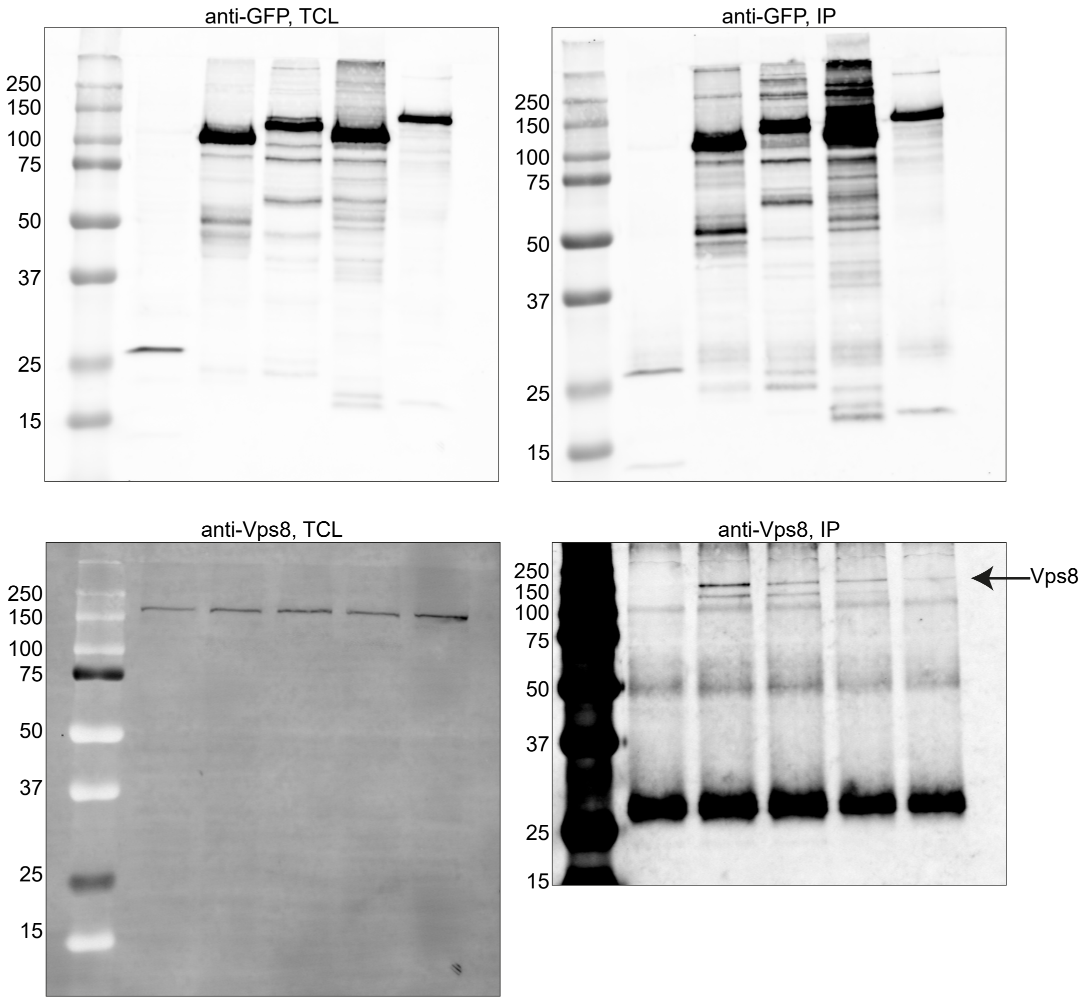

Supplementary Figure 2. Whole scans of blots from experiments of Figure 2a, molecular weights indicated, TCL= Total Cell Lysate, IP= Immunprecipitated.

Supplementary Figure 3

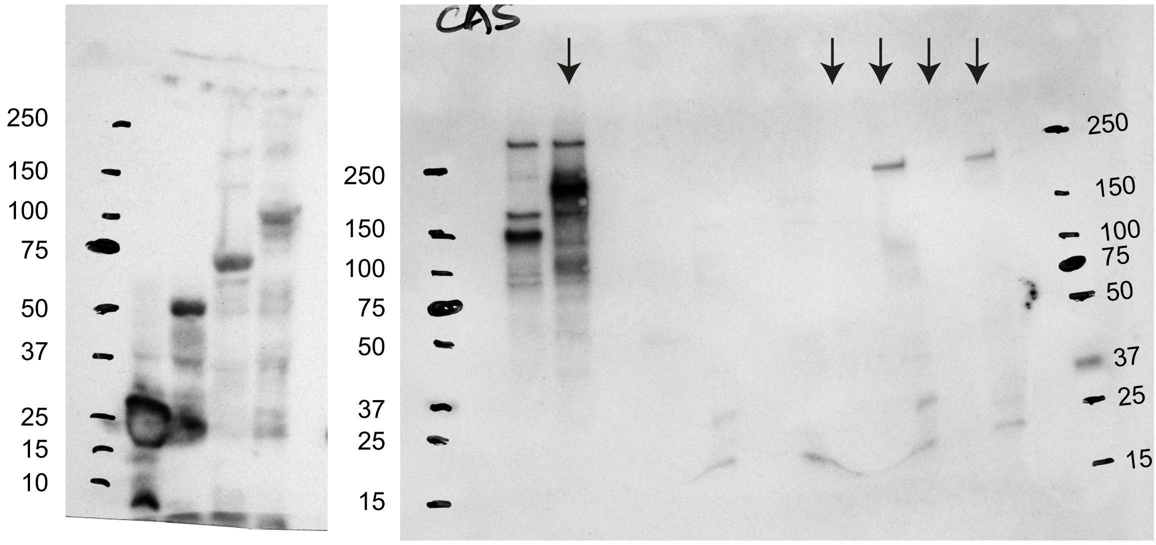

Supplementary Figure 3. Whole scans of blots from experiments of Figure 2b, molecular weights indicated, arrows indicate the lanes visible in Figure 2b.

Supplementary Figure 4

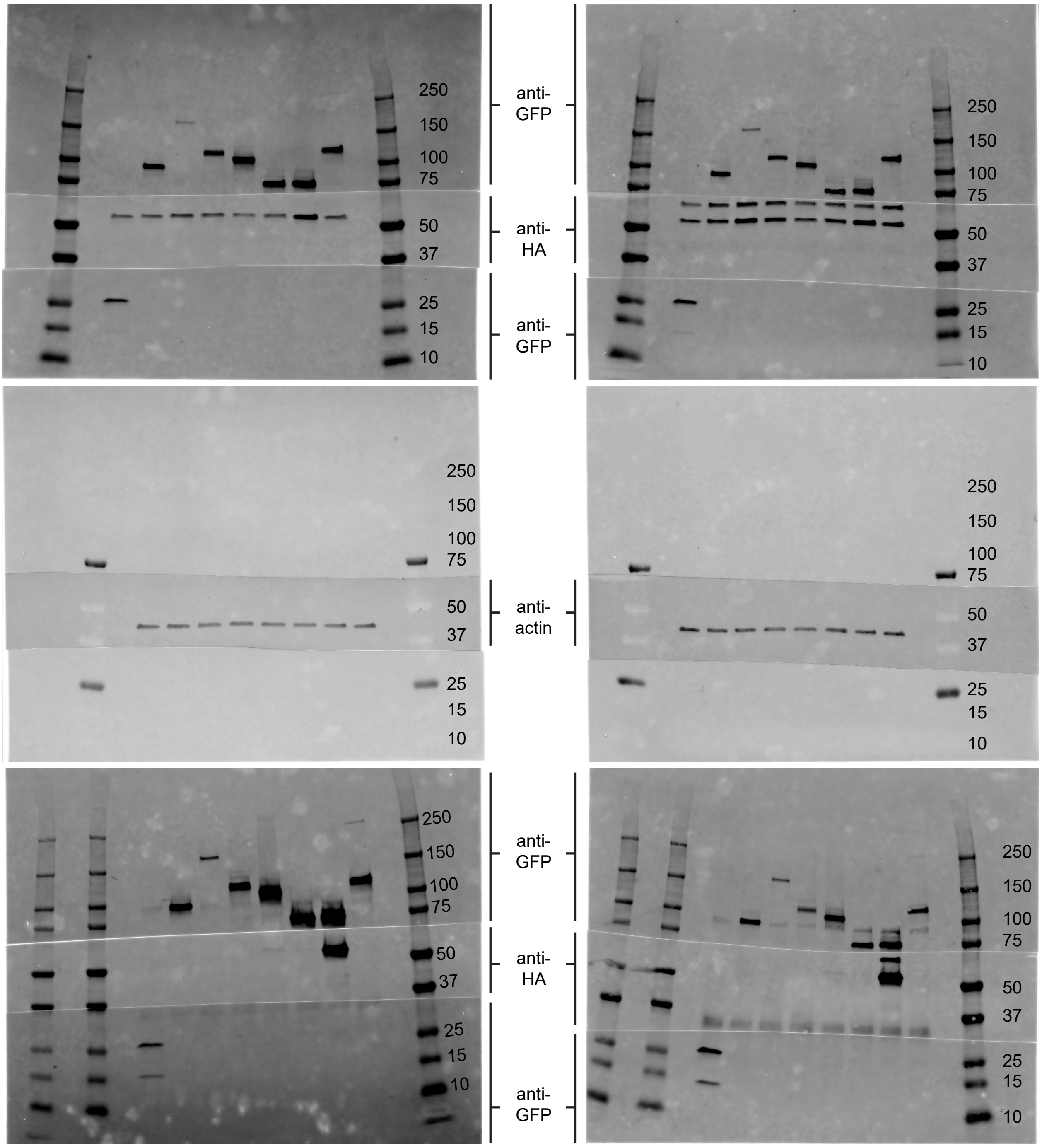

Supplementary Figure 4. Whole scans of blots from experiments of Figure 3d, molecular weights indicated, TCL= Total Cell Lysate, IP= Immunprecipitated.

Supplementary Figure 5

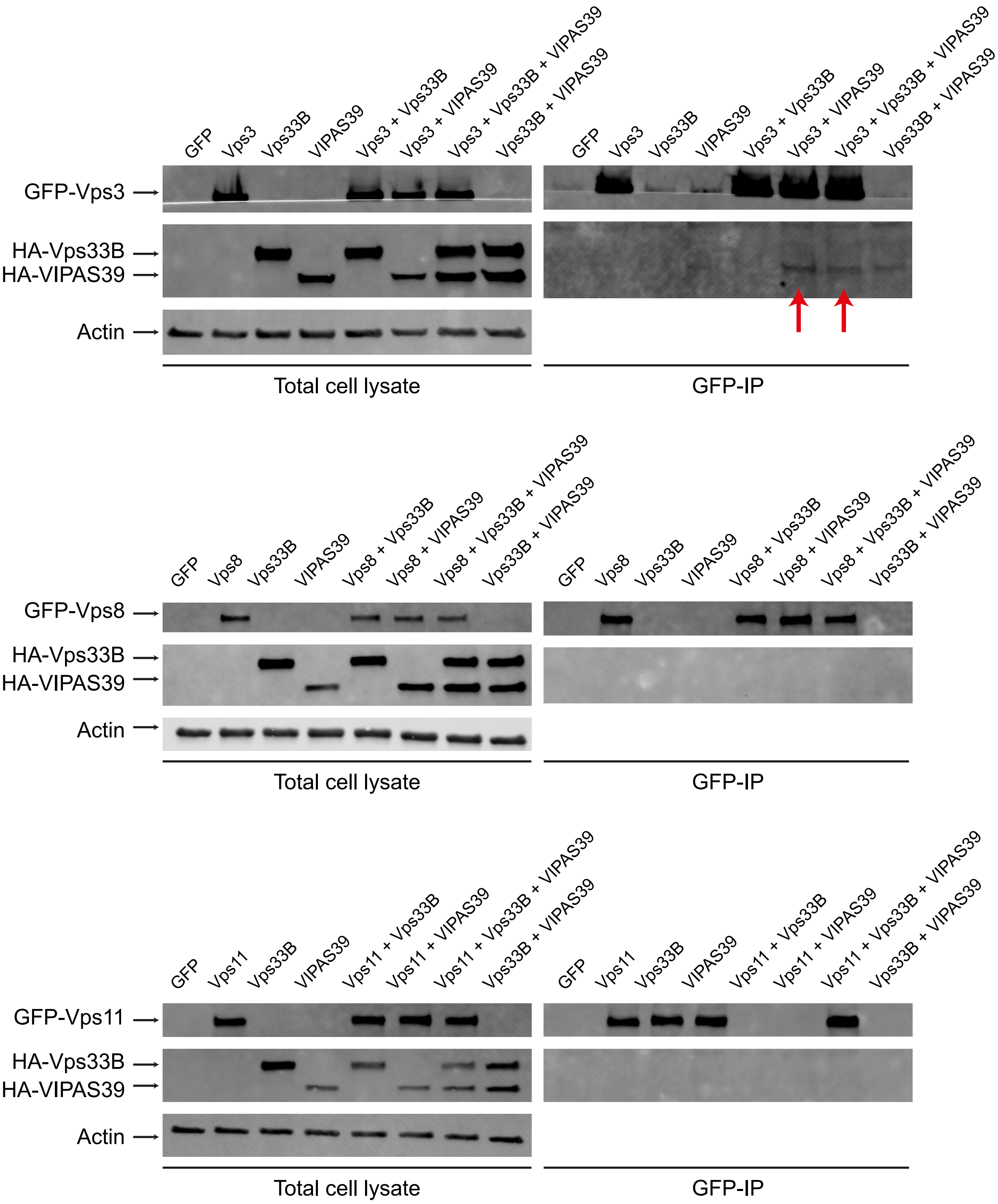

Supplementary Figure 5. IPs of GFP-tagged Vps3, Vps8 or Vps11 probed for interaction with co-expressed HA-VIPAS39 and/or HA-Vps33B in HeLa cells. Only Vps3 shows an interaction with VIPAS39, both in the presence or absence of Vps33B. This is a weak interaction compared to the interaction between Vps33B and VIPAS39 (fig. 3d).

Supplementary Figure 6

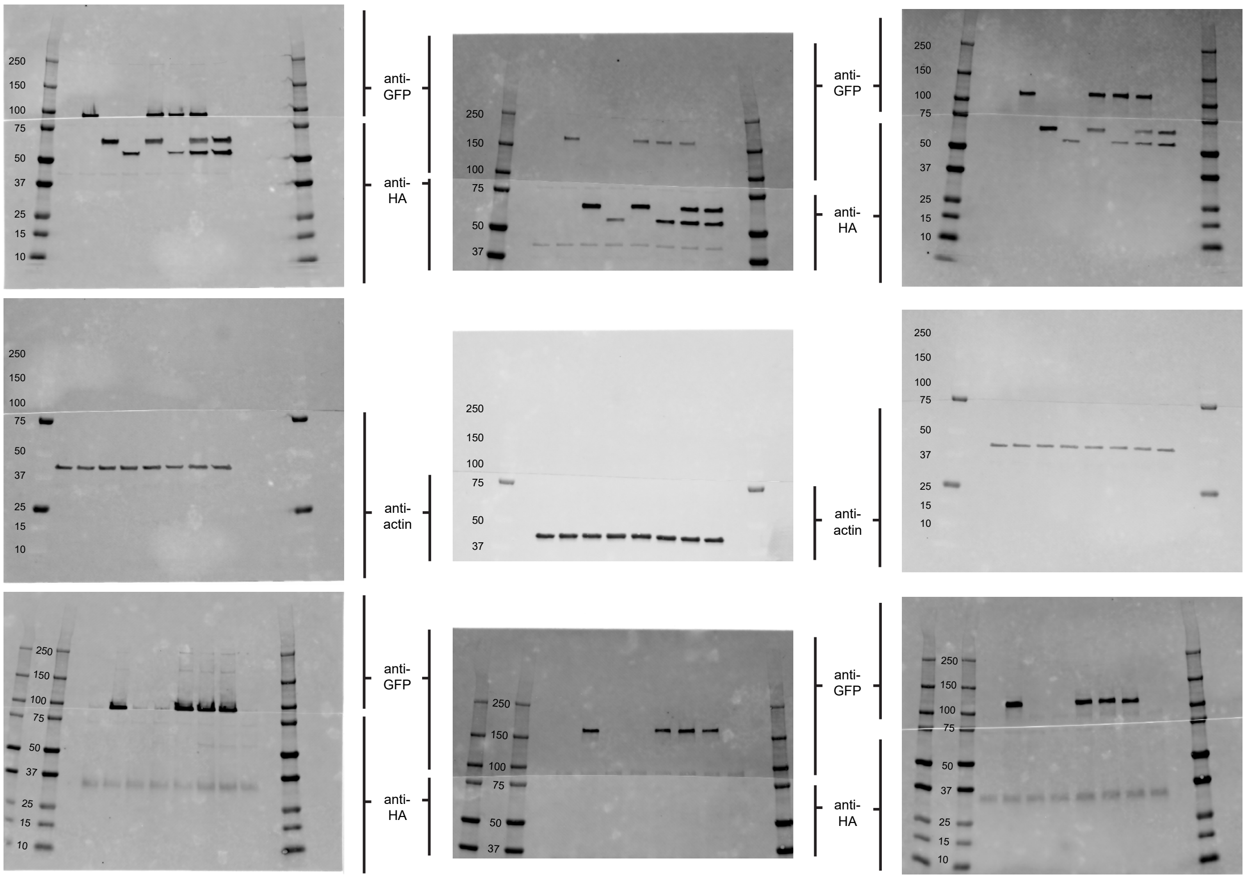

Supplementary Figure 6. Whole scans of blots from experiments of Supplementary Figure 5, molecular weights indicated, TCL= Total Cell Lysate, IP= Immunprecipitated.

Supplementary Figure 7

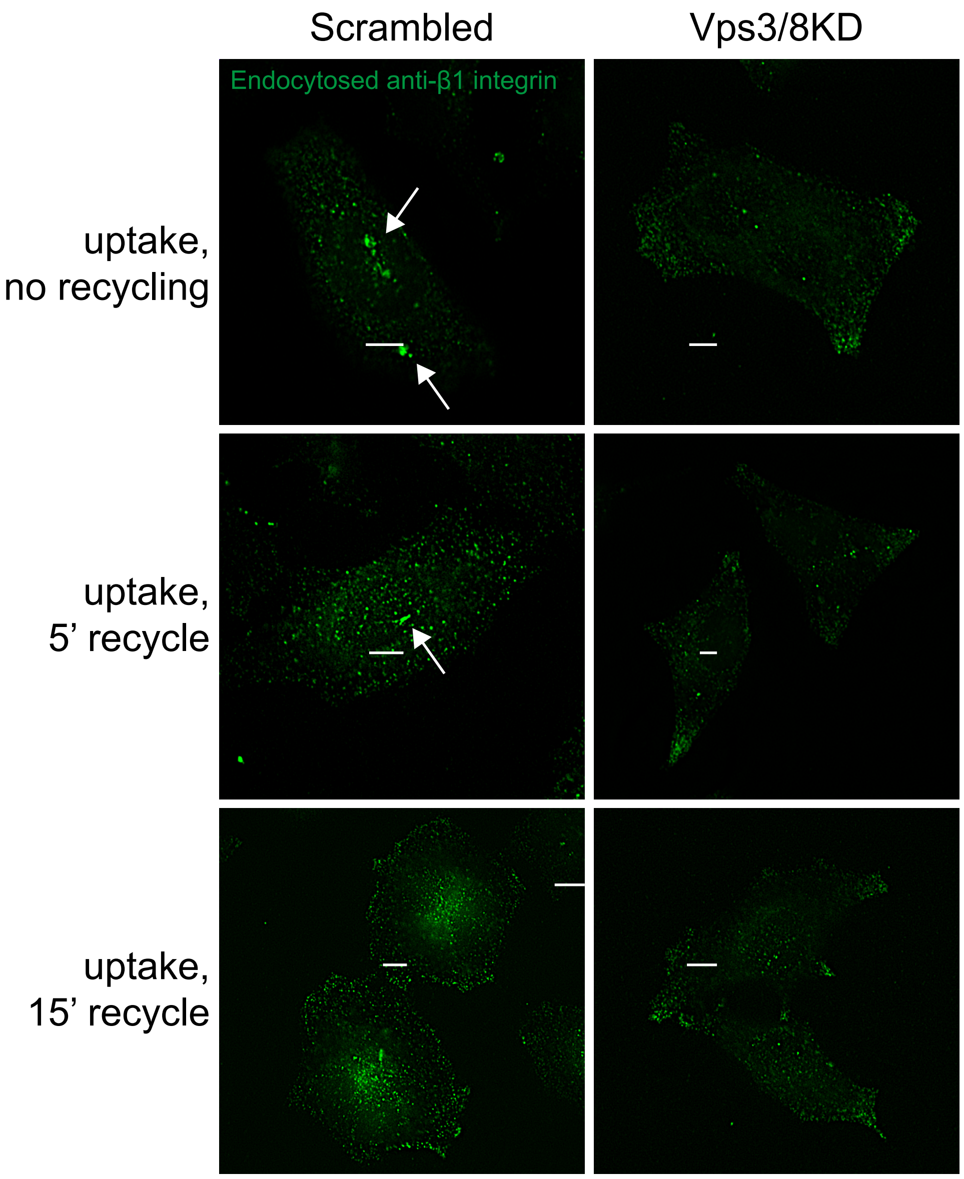

Supplementary Figure 7. Integrin recycling assay. HeLa cells knocked down for Vps3/8 were serum-starved overnight and subsequently incubated with anti-β1-integrin. After 2hr uptake at 37°C, and labelling with fluorescent secondary antibodies, β1-integrins accumulate in REs (arrows). After addition of serum, β1-integrins are rapidly recycled and the accumulations disperse. Shown are time points before addition of serum (upper panels) 5 minutes after addition of serum (middle panels) and 15 minutes after addition of serum (lower panels). In knockdown cells, no accumulation of internalised integrins is observed, indicating that trafficking to the REs is impaired. Bar, 10μm.

Supplementary Figure 8

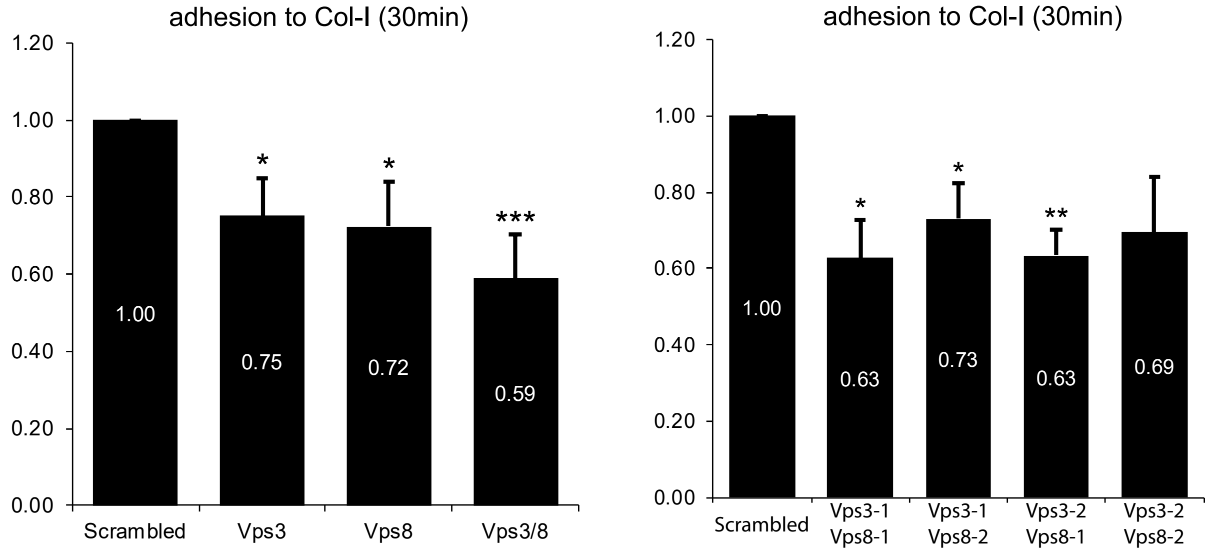

Supplementary Figure 8. (a) Knockdown of Vps3 or Vps8 separately affects cell adhesion less severely than the combined Vps3/8 knockdown. (b) Combinations of different single oligo's targeting Vps3 or Vps8 have the same effect on cell adhesion to Col-I (fig. 5b), showing that the adhesion defect is not due to off-target effects. Error bars represent SD. * = P ≤ 0.05, ** = P≤0.01, *** = P≤0.001 calculated using student's t-test.
